# Dissecting order amidst chaos of programmed cell deaths: construction of a diagnostic model for KIRC using transcriptomic information in blood-derived exosomes and single-cell multi-omics data in tumor microenvironment

**DOI:** 10.3389/fimmu.2023.1130513

**Published:** 2023-04-19

**Authors:** Chengbang Wang, Yuan He, Jie Zheng, Xiang Wang, Shaohua Chen

**Affiliations:** ^1^ Department of Urology, The First Affiliated Hospital of Guangxi Medical University, Nanning, China; ^2^ Guangxi Key Laboratory for Genomic and Personalized Medicine, Center for Genomic and Personalized Medicine, Guangxi Collaborative Innovation Center for Genomic and Personalized Medicine, Guangxi Medical University, Nanning, China; ^3^ Department of Urology, The Second Nanning People’s Hospital, Nanning, China; ^4^ Department of Urology, Shanghai General Hospital, Shanghai Jiao Tong University School of Medicine, Shanghai, China

**Keywords:** kidney renal clear cell carcinoma, programmed cell death, exosomes, single-cell RNA sequencing, spatial transcriptome, prognosis, biomarkers

## Abstract

**Background:**

Kidney renal clear cell carcinoma (KIRC) is the most frequently diagnosed subtype of renal cell carcinoma (RCC); however, the pathogenesis and diagnostic approaches for KIRC remain elusive. Using single-cell transcriptomic information of KIRC, we constructed a diagnostic model depicting the landscape of programmed cell death (PCD)-associated genes, namely cell death-related genes (CDRGs).

**Methods:**

In this study, six CDRG categories, including apoptosis, necroptosis, autophagy, pyroptosis, ferroptosis, and cuproptosis, were collected. RNA sequencing (RNA-seq) data of blood-derived exosomes from the exoRBase database, RNA-seq data of tissues from The Cancer Genome Atlas (TCGA) combined with control samples from the GTEx databases, and single-cell RNA sequencing (scRNA-seq) data from the Gene Expression Omnibus (GEO) database were downloaded. Next, we intersected the differentially expressed genes (DEGs) of the KIRC cohort from exoRBase and the TCGA databases with CDRGs and DEGs obtained from single-cell datasets, further screening out the candidate biomarker genes using clinical indicators and machine learning methods and thus constructing a diagnostic model for KIRC. Finally, we investigated the underlying mechanisms of key genes and their roles in the tumor microenvironment using scRNA-seq, single-cell assays for transposase-accessible chromatin sequencing (scATAC-seq), and the spatial transcriptomics sequencing (stRNA-seq) data of KIRC provided by the GEO database.

**Result:**

We obtained 1,428 samples and 216,155 single cells. After the rational screening, we constructed a 13-gene diagnostic model for KIRC, which had high diagnostic efficacy in the exoRBase KIRC cohort (training set: AUC = 1; testing set: AUC = 0.965) and TCGA KIRC cohort (training set: AUC = 1; testing set: AUC = 0.982), with an additional validation cohort from GEO databases presenting an AUC value of 0.914. The results of a subsequent analysis revealed a specific tumor epithelial cell of TRIB3^high^ subset. Moreover, the results of a mechanical analysis showed the relatively elevated chromatin accessibility of TRIB3 in tumor epithelial cells in the scATAC data, while stRNA-seq verified that TRIB3 was predominantly expressed in cancer tissues.

**Conclusions:**

The 13-gene diagnostic model yielded high accuracy in KIRC screening, and TRIB3^high^ tumor epithelial cells could be a promising therapeutic target for KIRC.

## Introduction

Renal cell carcinoma (RCC) is the most prevalent solid kidney lesion, accounting for 90% of renal malignancies (1) and 3% of all cancers (2). Kidney renal clear cell carcinoma (KIRC) is the most frequently diagnosed pathological classification, occupying about 80% of RCC (3). Despite the relatively favorable KIRC prognosis, with a 5-year survival rate of 75%, almost 30% of locally advanced cases will relapse with a locoregional recurrence or distant metastases (4, 5). The past decade has certainly witnessed remarkable advances in the characterization of KIRC management and research; nonetheless, much remains to be elucidated regarding the disease’s pathogenesis and underlying mechanism, and research into the identification of diagnostic approaches for KIRC is in its infancy. In this scenario, constructing a novel clinical model spanning screening, diagnosis, and prognosis predictions is of tremendous significance to clinical settings and provides novel insights into precision medicine therapeutic decisions.

In recent years, programmed cell deaths (PCDs) have generated holistic attention for researchers due to their inestimable potential in diagnostic biomarkers and therapeutic targets in cancer. Several PCD types have been identified, including apoptosis, necroptosis, autophagy, pyroptosis, ferroptosis, and cuproptosis, all considered cell-dependent and orderly cell death regulated by certain genes, with the purpose of homeostasis preservation and clearance of abnormal cells (6). PCDs are dynamically plastic, exert a dual role in distinct contexts and stages of cancer development (7), and are tightly regulated by spatiotemporal gene expression modulation. Unambiguous evidence suggests that KLF2 deficiency contributes to the suppression of ferroptosis and promotes the progression and metastasis of RCC cells (8). Similarly, Peng et al. demonstrated that silencing key autophagy-related genes could promote anoikis resistance and lung colonization of hepatocellular carcinoma (HCC) cells (9). Recent research advances and efforts in PCDs have predisposed to a significant growth in our understanding of the pathomechanisms of various cancer types, including KIRC. However, such studies have been hampered by a single PCD type or limitations in experimental approaches, which obscure the subtle yet essential regulatory mechanisms underlying the surface.

Encouragingly, the emergence of blood-derived exosomes provides a new perspective on the mechanisms of cellular interactions in the tumor microenvironment (TME) and the search for tumor diagnostic biomarkers. Exosomes are cell-derived nano-vesicles, ranging from 30 to 150 nm in diameter, that transfer RNA, proteins, lipids, and metabolites to recipient cells in the body (10). Initially, exosomes were thought to be the inert debris produced by cells to dispose of wastes. As the study of exosomes deepened, it was gradually discovered that they are involved not only in antigen presentation, cell differentiation, and immune response but also in tissue inflammation, virus transmission, migration, and tumor cell invasion (11–13). A study by Zhang et al. found that the exosomal miR-522 secreted by cancer-associated fibroblasts inhibited ferroptosis in cancer cells by targeting ALOX15 and compromising lipid peroxide accumulation (14). Moreover, a study by Shen et al. reported that exosomes secreted by pancreatic cancer cells were taken up by T lymphocytes, which activated p38 MAPK and then induced endoplasmic reticulum stress-mediated apoptosis, ultimately causing immunosuppression (15). The abovementioned studies demonstrate the intimate association between PCDs and exosomes in TME. Existing studies, however, were conducted in biological assays devoid of a cellular microenvironmental context, which may result in unduly artificial outcomes. The link between PCDs and exosomes in the TME of KIRC is poorly understood as are the regulating processes.

The emergence of single-cell RNA sequencing (scRNA-seq) technology can partially solve the abovementioned problems. As a high-resolution tool, it overcomes the limitations of traditional bulk sequencing. It enables a breakthrough in the problem of exosomal mRNA traceability at the single-cell level by combining single-cell assays for transposase-accessible chromatin sequencing (scATAC-seq) and spatial transcriptomics sequencing (stRNA-seq) to study epigenetic regulation and observe the spatial distribution of key genes at single-cell resolution, synergistically uncovering molecular mechanisms at higher levels.

**Figure 1:**
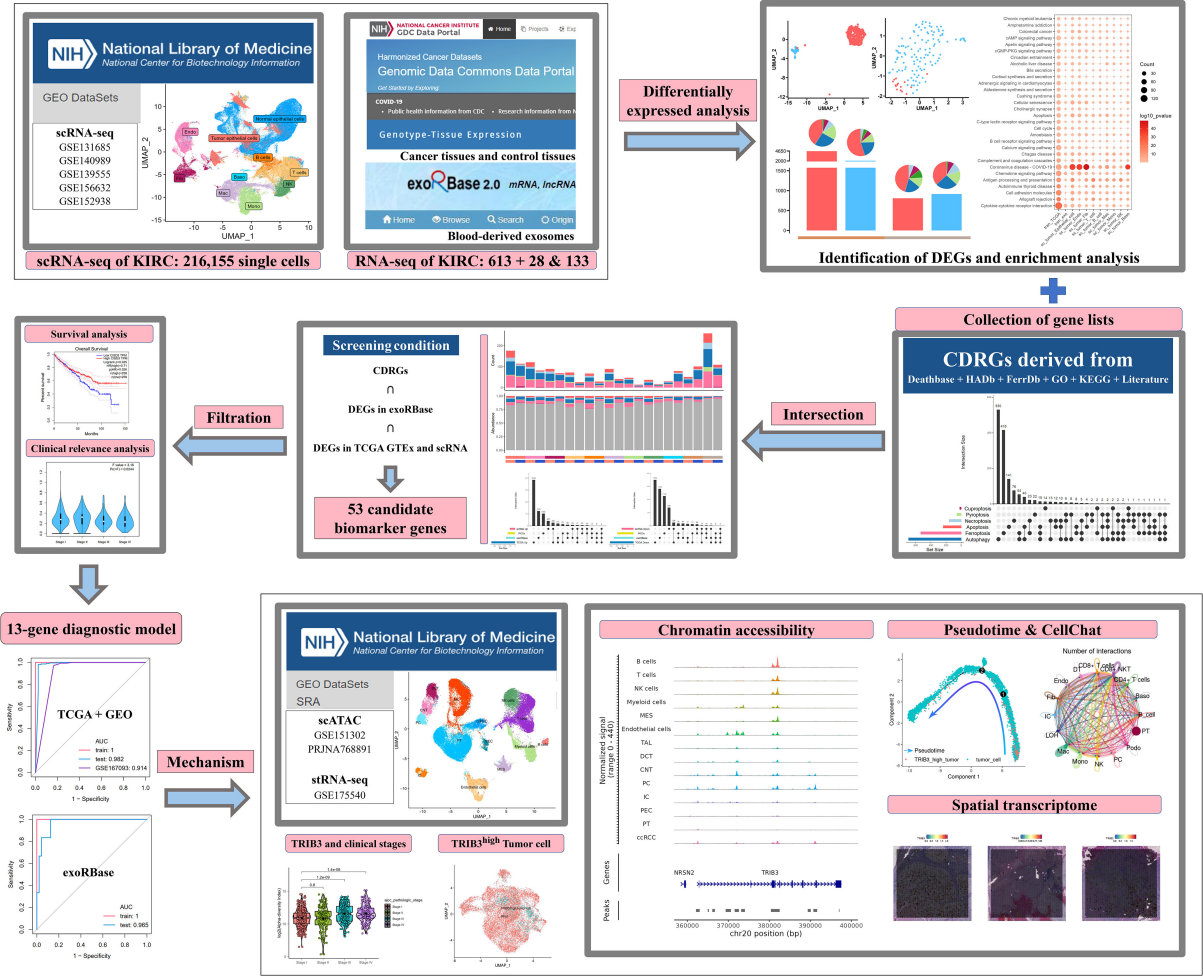
The dataset information and workflow of the presented study.

In the present study, we collected PCD-related genes, specifically (CDRGs), along with scRNA data and the KIRC cohort from The Cancer Genome Atlas (TCGA) database, to investigate the relationship between KIRC and PCD development. Meanwhile, we deciphered the blood-derived exosome transcriptome data to construct a gene model for clinical diagnosis and validated the diagnostic efficacy in KIRC cohorts by machine learning methods. Finally, we explored the mechanisms of these genes in the KIRC progression by scATAC data and cellular interaction network analysis. The abovementioned results support the clinical diagnosis and treatment decisions in KIRC. The dataset information and workflow of the presented study are shown in **Figure 1**.

## Materials and methods

### Acquisition of gene lists and multi-omics datasets

Six PCD categories were included, and their respective related genes, namely CDRGs, were collected. Among these, apoptosis- and necroptosis-related genes were collected from Deathbase (http://deathbase.org/), comprising proteins and corresponding coding genes of typical PCDs. Autophagy-related genes were collected from Human Autophagy Database (HADb; http://www.autophagy.lu). Ferroptosis- and pyroptosis-related genes were collected from Ferroptosis Database (FerrDb; http://www.zhounan.org/ferrdb) and published literature (16). Moreover, Gene Ontology (GO) and Kyoto Encyclopedia of Genes and Genome (KEGG) databases were also used to extract the associated genes in the PCDs mentioned above. The pyroptosis-related genes were derived from the GO database and published literature (17), while the cuproptosis-related genes were only obtained from published literature (18). Details of the CDRGs are listed in **Supplementary Table S1**.

A total of 11 independent datasets were included in this study, containing eight single-cell datasets, two bulk RNA-seq datasets of tissues, and bulk RNA-seq datasets of blood-derived exosomes. Data from eight single-cell datasets, included five scRNA-seq data of KIRC, para-carcinoma, and healthy tissues from nephrectomy or biopsies, are shown below: GSE131685 (*n* = 3), GSE140989 (*n* = 24), GSE139555 (*n* = 6), GSE156632 (*n* = 12), and GSE152938 (n = 3); one spatial transcriptome dataset of KIRC was derived from the GEO database with accession number GSE175540; two datasets contained five scATAC healthy kidney data and three scATAC KIRC data, which were downloaded from GSE151302 and the National Center for Biotechnology Information Sequence Read Archive under accession number PRJNA768891, respectively. Meanwhile, two bulk RNA-seq data of tissues of the TCGA-KIRC cohort with associated clinical information (*n* = 613) were downloaded from the TCGA (https://portal.gdc.cancer.gov/) databases, combined with the normal kidney tissue data downloaded from the GTEx portal (www.gtexportal.org). The other bulk transcriptomic data of KIRC cohort provided by the GEO database was used as an additional validation cohort with accession number GSE167093 (*n* = 656); one bulk transcriptomic data of blood-derived exosome was downloaded from the exoRbase database (http://www.exorbase.org/, *n* = 133).

### scRNA-seq data analysis

Fastq files were processed using Cell Ranger (version 6.1.2, 10x Genomics) with default parameters and mapped to 10x human transcriptome GRCh38-2020 (https://support.10xgenomics.com/single-cell-gene-expression/software/downloads/latest). Seurat (version 4.2.0) was used to process single-cell data for the following analyses. We filtered out low-quality cells with less than 400 or more than 5,000 total genes expressed or with more than 30% mitochondrial RNA contents. SCTransform, RunPCA, and RunUMAP functions were used for normalization and dimensionality reduction, respectively (19). In addition, harmony (version 0.1.1) was used to correct batch effects between different arrays (20). FindNeighbors and FindClusters functions were then used to differentiate the cell clusters with the dimensions and resolution parameters of 1:25 and 0.8, respectively. scHCL (version 0.1.1), SingleR (version 1.10.0), and ScType (https://github.com/IanevskiAleksandr/sc-type) packages were used to aid in the identification of cell subpopulations, and cluster-specific marker genes were identified by the FindAllMarkers function of Seurat package (logfc.threshold = 0.25, min.pct = 0.1).

### scATAC-seq data analysis

scATAC-seq was processed by Cell Ranger -atac-2.1.0 using default parameters and mapped to 10x human transcriptome GRCh38-2020 (https://support.10xgenomics.com/single-cell-gene-expression/software/downloads/latest). Signac (version 4.2.0, 10x Genomics) was used to analyze the output of the Cell Ranger ATAC pipeline. Low-quality cells were removed based on the following criteria: nucleosome signal score of less than 4 and transcriptional start site enrichment score of more than 3. RunTFIDF function was used for normalization, while RunSVD and RunUMAP were used for linear and nonlinear dimensional reductions, respectively (21). harmony (version 0.1.1) was likewise used to correct batch effects between arrays (20). Gene activity was quantified *via* the GeneActivity function in Signac, including the 2 kb upstream of the transcriptional start site and gene body.

### stRNA-seq data analysis

stRNA data was analyzed through Seurat (version 4.2.0). Spatial spots featuring less than 300 genes or more than 30% of mitochondrial genes were filtered out. Raw counts were normalized with the SCTransform function of Seurat with the assay of the spatial parameter. RunPCA and RunUMAP functions were used for dimensionality reduction.

### Bulk RNA-seq data processing

We used stringr (version 1.4.1) and stats (version 4.2.1) in R language to integrate the data of KIRC dataset from the TCGA database and the control samples from the GTEx database as well as the raw data matrix of KIRC downloaded from the GEO database. The data were collated and filtered under the following conditions: (1) genes detected in all samples were retained, (2) genes with sum of counts across all samples less than 2.5 were excluded from further analyses, (3) genes with an average expression higher than 0 in at least 80% of the tumor or control samples were retained, (4) the expression levels of duplicated genes in the data matrix were averaged, and (5) batch effects between the TCGA and GTEx databases were corrected using the ComBat function from sva (version 3.44.0) package.

### Identification of differentially expressed genes

Differential gene expression analysis in single-cell datasets was performed using the FindMarkers function in the Seurat package with *P*-value <0.05 and |log_2_FC| >0.25 as cutoff criteria. DESeq2 (version 1.36.0), limma (version 3.52.4), and edgeR (version 3.38.4) packages were used for the identification of DEGs in the TCGA KIRC cohort, with P-value <0.05 and |log_2_FC| >1 as the thresholds. In the bulk RNA-seq data of blood-derived exosomes, differentially expressed genes (DEGs) were recruited using |log_2_FC| >0.5 and *P*-value <0.05. The intersection analysis of DEGs between different datasets was visualized using the UpSetR (version 1.4.0) package. We then used ggplot2 (version 3.3.6) to visualize the expression differences and expression of key genes by means of bubble plots and heat maps.

### Gene Ontology analysis and Kyoto Encyclopedia of Genes and Genomes analysis

GO function enrichment analysis and KEGG pathway enrichment analysis of the target genes in RNA-seq were performed using R package clusterProfiler (version 4.4.4). The results were filtered with a *P*-value of 0.05.

### Correlation analysis between the target genes and clinical parameter

ggpubr (version 0.4.0) package was loaded to perform the correlation analysis of target genes with clinical parameters using the stat_compare_means function, thereby visualizing data with boxplots using ggplot2 package. GEPIA2.0 (http://gepia2.cancer-pku.cn/#index, accessed on December10, 2022), a platform for TCGA data visualization, was also utilized to evaluate the effect of candidate biomarker genes on overall survival in KIRC and to create Kaplan–Meier survival curves. It was also used to analyze the correlations between candidate biomarker genes and clinical indicators.

### Machine learning analysis

We used stratified random sampling to divide exoRBase KIRC into a training set and a testing set in a ratio of 3:2. The training set was used to construct the random forest classification model, and the testing set was used to validate the model further. The constructed model’s performance was assessed by calculating the area under the curve (AUC) value. The same approach was used for the TCGA KIRC cohort merged with GTEx samples to observe the diagnostic efficacy of key genes in the tissue. The abovementioned process was performed using the tidymodels (version 1.0.0) and pROC (version 1.18.0) R packages.

### Cell–cell interaction network analysis

Intercellular interaction analysis was conducted using CellChat (version 1.5.0) (22), based on which we could identify the potential ligand–receptor interactions according to the expression pattern of ligands in one cell subtype and their corresponding receptors in the other cell subtypes.

### Reconstructing TRIB3^high^ tumor cell differentiation trajectories by Monocle2

Fate decisions and pseudotime trajectories of TRIB3^high^ tumor cells were reconstructed using the Monocle2 R package (version 2.24.1). First, tumor epithelial cells were selected by Seurat, and 16,747 tumor cells were imported into Monocle2 with a lower detection limit parameter of 0.5. Subsequently, we performed differential gene expression analysis using the differentialGeneTest function and retained DEGs with q-value <0.01 as sorted gene sets and performed descending dimensionality and trajectory analysis. We finally determined the direction of the cell differentiation trajectory by the cell stemness-related gene CD44 and visualized the trajectory results using the plot_cell_trajectory function.

### Statistical analysis

The categorized variables between groups were compared using Wilcoxon test, and a correlation analysis between different cell subtypes was performed using Spearman correlation test. A *P*-value less than 0.05 was considered to indicate statistical significance. R language (version 4.2.1; http://www.r-project.org/) was used for data analyses and figure generation unless indicated otherwise.

## Results

### Transcriptome information of KIRC in multiple tissue sources

**Figure 2:**
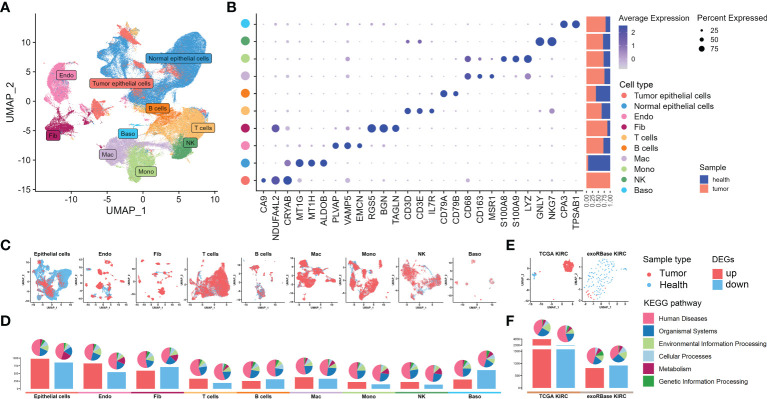
Single-cell RNA sequencing (scRNA-seq) and bulk RNA sequencing (RNA-seq) profiling of kidney renal clear cell carcinoma (KIRC). **(A)** Uniform manifold approximation and projection showing the 10 major cell clusters in the scRNA-seq datasets. **(B)** Marker genes and proportions of sample origins for the 10 major cell clusters of the scRNA-seq datasets. **(C)** Distribution characteristics of the 10 major cell clusters in the scRNA-seq datasets. **(D)** Barplots showing the counts of differentially expressed genes (DEGs) between the cancer and control samples of each cell cluster in the scRNA-seq datasets. **(E)** Distribution characteristics of The Cancer Genome Atlas (TCGA) KIRC cohort merged with control cases from the GTEx database (left) and exoRBase KIRC cohort (right) containing the RNA-seq data of blood-derived exosomes of patients. **(F)** Barplots showing the counts of DEGs between the KIRC and healthy cases of TCGA KIRC and exoRBase KIRC cohorts. The pie plots at the top of the bar show the Kyoto Encyclopedia of Genes and Genomes pathway enriched by each group of DEGs.

We started our investigations with the KIRC expression profiles at single-cell resolutions. We assembled 48 KIRC cases from five independent datasets provided by the GEO database. These were containing cancer, para-carcinoma, and healthy tissues from nephrectomy or biopsies. After the implementation of stringent quality control, 216,155 single cells from five independent datasets were retained for the following analyses. The sample information and quality control data are shown in **Supplementary Table S2** and **Supplementary Figures S1A, B**. Having processed with the Seurat package and removed the batch effect, 54 cell clusters (**Supplementary Figures S2A–C**) and 10 main cell types were identified, including tumor epithelial cell, normal epithelial cell, endothelial cell (Endo), fibroblast (Fib), T cell, B cell, macrophage (Mac), monocyte (Mono), natural killer cell (NK), and basophil (Baso), thus visualized through uniform manifold approximation and projection (UMAP) (**Figure 2A**). The marker genes of each cell cluster are shown in **Supplementary Table S3**. The specific markers and relative abundance for the main cell types are shown in **Figure 2B**. Specifically, epithelial cells dominated all major cell compartments, with tumor epithelial cells expressing the canonical markers of CA9 coming exclusively from tumor tissues and normal epithelial cells having multiple origins. The distributions for each main cell type and their origins were visualized using UMAP (**Figure 2C**). Subsequently, we explored the DEGs between the cancer and control samples of various major cell types based on their expression profiles (**Supplementary Table S4**), with the bar plots indicating the exact counts of upregulated and downregulated DEGs and the pie plots manifesting their corresponding categories in the KEGG pathways (**Figure 2D**), most of which belong to “human disease”. Intriguingly, the highest DEG number was presented between tumor and normal epithelial cells, followed by DEGs between Endo and Fib between cancer and control samples (**Figure 2D**), demonstrating the dramatic alterations of structural cells in transcriptome and their essential stages in tumorigenesis.

Next, we further dissected the transcriptome landscape of KIRC based upon the TCGA cohort merged with healthy samples in GTEx (**Figure 2E**), revealing 4,604 upregulated and 2,073 downregulated DEGs in cancer tissues (**Figure 2F**; **Supplementary Table S5**). An increasing body of unambiguous evidence denotes the role of cancer cell-derived exosomes of patients on the course of epithelial–mesenchymal transition and metastasis in KIRC (23, 24), thus making it a promising diagnostic and prognostic KIRC biomarker. As such, we then analyzed the RNA-seq data of human blood-derived exosomes of healthy controls and KIRC patients using the exoRBase database, with UMAP showing complete separations between cancer and control samples (**Figure 2E**), and the differential gene expression analysis yielded a total of 1,723 DEGs (**Figure 2F**; **Supplementary Table S6**). Notably, the KEGG functional enrichment analyses between groups elucidated that such DEGs were mainly enriched in cell cycle, apoptosis, cancer, and immune-related signaling pathway (**Supplementary Figures S3A, B**). In a nutshell, we investigated the transcriptome data of tissue- and blood-derived exosomes of KIRC patients and corresponding controls exhaustively, combining them with DEGs of various cell types based on scRNA data, thereby laying the groundwork for a subsequent analysis to identify disease biomarkers.

### The expressed pattern of CDRGs in KIRC

**Figure 3:**
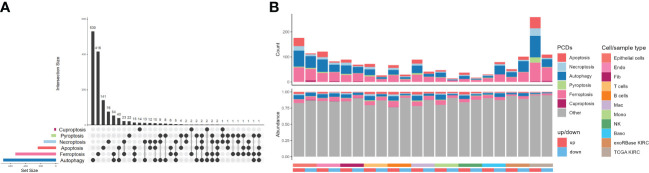
Distribution characteristics of programmed cell deaths in kidney renal clear cell carcinoma (KIRC). **(A)** UpSet plot showing the intersection analysis of the six types of cell death-related genes (CDRGs). **(B)** Distribution characteristics of the differentially expressed genes (DEGs) in single-cell RNA sequencing, The Cancer Genome Atlas KIRC, and exoRBase KIRC cohorts and their shared genes with six classes of CDRGs. The top bar plot represents the counts of DEGs shared CDRGs, and the bottom bar plot shows the relative abundance of CDRGs in DEGs of all the groups.

As planned, six kinds of PCDs, including apoptosis, necroptosis, autophagy, pyroptosis, ferroptosis, and cuproptosis, and their related genes, namely CDRGs, showing commonalities and specificities were included (**Figure 3A**). However, we were unable to uncover any genes that were shared by all PCD categories, but the number of genes shared by ferroptosis and autophagy was very high. As depicted in **Figure 3B**, most CDRGs were upregulated DEGs in single-cell and RNA-seq datasets. However, the proportions of downregulated DEGs were very low in such datasets, with most of them belonging to the autophagy, apoptosis, and ferroptosis pathways. Such a phenomenon raised an illustrative assertion: the expression levels of CDRGs were enhanced in varying degrees. Notwithstanding, this explicit demonstration of the eye-catching alterations of such genes implicitly proposed the question of what specialized roles they played in TME.

**Figure 4:**
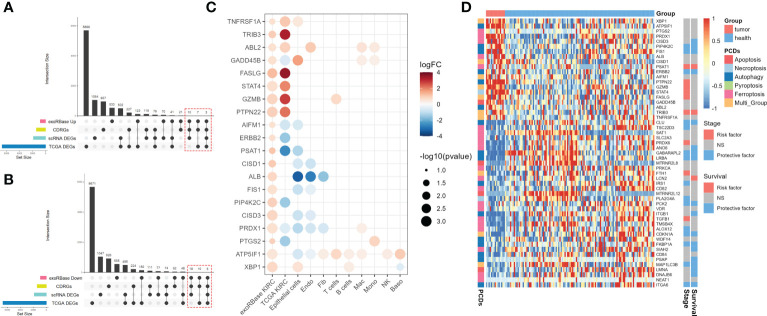
Screen for candidate biomarker genes. **(A)** UpSet plots showing the intersection analysis among cell death-related genes (CDRGs), differentially expressed genes (DEGs) in exoRBase kidney renal clear cell carcinoma (KIRC) cohort, upregulated DEGs in The Cancer Genome Atlas (TCGA) KIRC cohort, and scRNA datasets. **(B)** UpSet plots showing the intersection analysis among CDRGs, DEGs in the exoRBase KIRC cohort, downregulated DEGs in the TCGA KIRC cohort, and scRNA datasets. **(C)** The bubble plots show 20 candidate biomarker genes upregulated in the exoRBase KIRC cohort and their expression pattern in other datasets. Red circles represent positive logFC values or upregulated DEGs in corresponding datasets, while blue circles represent negative logFC values or downregulated DEGs in corresponding datasets; the bubble size indicates negative log10(*P*-value). **(D)** Heat map showing the expression levels of 53 candidate biomarker genes in the exoRBase KIRC cohort, with red color indicating relatively high expression and blue color indicating relatively low expression levels. The column annotations on the left side represent the programmed cell death classification of the candidate biomarker genes. The two annotated columns on the right side show the correlation of candidate biomarker gene expression with the survival outcome and clinical stage of the TCGA KIRC cohort, respectively. Red color represents the gene as a risk factor, and blue color represents a protective factor in the prognosis of KIRC cases.

Next, we intersected the upregulated (**Figure 4A**) and downregulated (**Figure 4B**) DEGs in exoRBase KIRC with CDRGs and DEGs obtained from single-cell and TCGA datasets to screen for the candidate biomarker genes. Herein we retained the differentially expressed CDRGs between the exoRBase and TCGA databases or differentially expressed CDRGs between the exoRBase and single-cell datasets, thereby acquiring 53 candidate biomarker genes. Notably, 20 genes were upregulated (**Figure 4C**), and 33 genes were downregulated in the blood-derived exosomes of KIRC patients (**Supplementary Figure S4A**). Concomitantly, such differentially expressed trends of candidate biomarker genes were largely consistent in the TCGA datasets and single-cell datasets of epithelial cells, Endo, and Fib, namely structural cells. Such discoveries denoted the pivotal role of exosomes in orchestrating the dialog with neoplastic cells and profoundly influencing the TME alteration.

Following are the correlations between 53 candidate biomarker genes and clinical markers. The results indicated that 32 out of 53 genes were closely associated with patients’ clinical stages or survival outcomes, functioning doubly as a risk or protective factor in KIRC (**Figure 4D**). Moreover, ferroptosis- and autophagy-related genes account for 32 genes, with a small proportion of genes belonging to apoptosis and necroptosis. Thereinto, 13 out of 32 genes were simultaneously related to the clinical stages and survival outcomes of KIRC (**Supplementary Figure S4B** and **Supplementary Figure S5**), including PIP4K2C, FIS1, PSAT1, ERBB2, TRIB3, CLU, GABARAPL2, LRBA, PCK2, CDKN1A, FKBP1A, MAP1LC3B, and ITGA6, which are subject to the following analysis.

PSAT1, a risk factor in KIRC, had contradictory expression patterns in blood-derived exosomes and tissues, with the former displaying an elevated expression level and the latter displaying a downregulated expression level. Such phenomena are reminiscent of the connections between exosome releasing and signaling reception of neoplastic cells, possibly contributing to the alteration of expression profiles in TME and the emergence of malignant cancer phenotypes (25). In summary, we comprehensively explored the CDRG expression pattern in KIRC, based on which we carried out the correlation analyses of candidate marker genes with clinical indicators, identifying 13 key genes linked with survival outcomes and clinical stages of KIRC cases.

### Validation of the 13-gene diagnostic model and mechanism explorations

**Figure 5:**
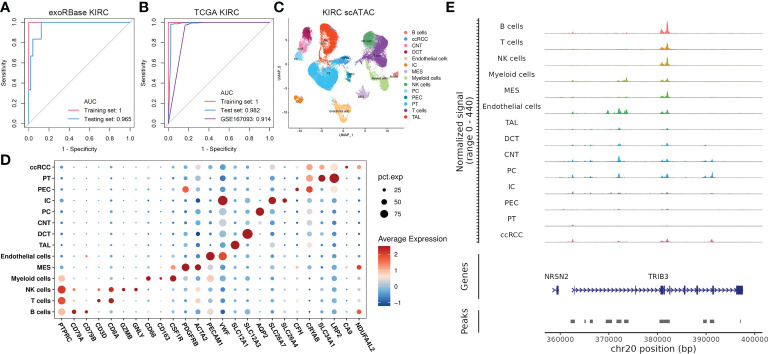
Construction of 13-gene diagnostic models and single-cell assays for transposase-accessible chromatin (scATAC-seq) analysis. **(A)** Receiver-operating characteristic (ROC) curve analysis of a 13-gene diagnostic model based on exoRBase kidney renal clear cell carcinoma (KIRC) cohort, with the red curve representing the training set and the blue curve representing the testing set. **(B)** ROC curve analysis of a 13-gene diagnostic model based on The Cancer Genome Atlas KIRC combined with GTEx cohort and KIRC cohort from the GEO database with accession number GSE167093, with the former treated as the training set (red) and the testing set (blue). In contrast, the latter was an additional validation cohort (purple). **(C)** Uniform manifold approximation and projection plot showing the 14 cell clusters in the scATAC-seq analysis **(D)** Bubble plots showing the marker genes for each cell cluster in scATAC-seq. **(E)** CoveragePlot showing the peak–gene links for TIRB3.

Next, we used 13 key genes to construct a diagnostic model for KIRC, as previously described in the “Materials and methods”. Specifically, we randomly stratified all samples from the exoRbase database into two groups (training set and the testing set) with a ratio of 3:2 for cross-validation. Encouragingly, the 13-gene diagnostic model presented outstanding discriminatory ability in the KIRC datasets of the exoRbase database (**Figure 5A**), with AUC values of 1 and 0.965 in the training and testing sets, respectively. Similarly, the model constructed with 13 genes in the TCGA KIRC cohort showed promising diagnostic results, with AUC values of 1 and 0.982 in the training and testing sets, respectively (**Figure 5B**). Furthermore, GSE167093, provided by the GEO database containing 656 KIRC cases, was used as an additional validation cohort, exhibiting a tremendously high diagnostic accuracy with an AUC value of 0.914. The findings unequivocally demonstrated that the 13-gene diagnostic model was very stable and trustworthy in detecting KIRC, regardless of whether the sample was taken from blood-derived exosomes or solid tissue, and ensured high sensitivity and specificity. Moreover, the differentially expressed trend in blood-derived exosomes may provide an instant advantage in liquid biopsy analyses for biomarker evaluations, reducing the sampling inconveniences and hazards.

Nevertheless, the molecular basis for the 13-gene diagnostic model has not been addressed. Such combinations of genes derived from the transcriptomic data of exosomes and various cell subtypes are not as simple as they may seem. The crosstalk behind the cellular identities and their exosomes confers intriguing information about the KIRC pathogenesis. Thus, we then focused on studying the epigenetic profile of KIRC in scRNA and scATAC data to uncover the role of such genes in transcriptome and epigenetic regulation at single-cell resolutions. We discovered the abnormal expression pattern of such genes in distinct cell types based on scRNA data, especially for CLU, CDKN1A, PSAT1, and MAP1LC3B, which are differentially expressed in virtually all cell types (**Supplementary Figure S6**). Then, we analyzed 63,489 cells in the scATAC datasets of KIRC cases, identifying 15 main cell types based on the average promoter activity of representative marker genes (**Figures 5C, D**). Of particular interest is the fact that we found that TRIB3 expression was higher in tumor epithelial cells referred to normal epithelial cells. At the same time, its chromatin accessibility was significantly increased compared with the normal PT cell cluster (**Figure 5E**), a common type of epithelial cell in the kidney.

### Comprehensive descriptions of TRIB3^high^ tumor epithelial cells

**Figure 6:**
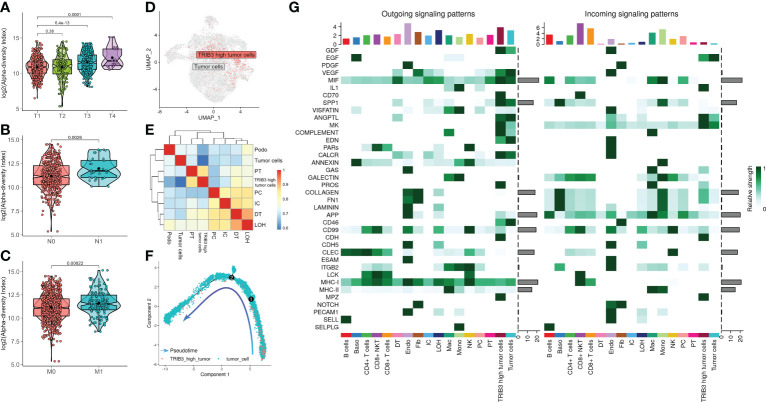
Clinical characteristics of TRIB3 expression in kidney renal clear cell carcinoma (KIRC) patients and characterization of the TRIB3^high^ tumor cell subset. **(A–C)** The box plot shows the correlation between TRIB3 expression and T classification, N classification, and M classification in KIRC patients. **(D)** Uniform manifold approximation and projection plot indicating the distribution pattern of TRIB3^high^ tumor cells. **(E)** Heat map showing the correlation between various cell types using the Spearman method; the colors represent the strength of the correlation. **(F)** Pseudotime analysis of TRIB3^high^ tumor cells. The direction of the arrow indicates the differentiation trajectory. **(G)** Signaling role analysis showing the aggregated cell–cell communication networks from all signaling pathways. The shades of color represent the relative strength of cellular communication.

**Figure 7:**
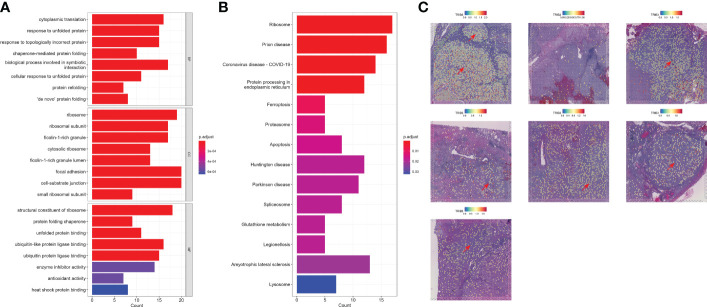
Functional analysis and spatial localization of TRIB3^high^ tumor cells. **(A)** Gene Ontology enrichment analysis for upregulated differentially expressed genes (DEGs) in TRIB3^high^ tumor cells. **(B)** Kyoto Encyclopedia of Genes and Genomes pathway analysis for upregulated DEGs in TRIB3^high^ tumor cells. **(C)** Visualization of TRIB3^high^ tumor cells in kidney renal clear cell carcinoma spatial transcriptome tissue sections.

Next, we investigated the TRIB3 influence in TME and its corresponding cell subset. The subsequent analysis of TRIB3 demonstrated that this gene was positively associated with TNM staging of KIRC (**Figures 6A–C**), implying its adverse role in the survival outcome of KIRC, which could be the leading contributor to the metastasis of cancer cells. Therefore, our analysis focused on understanding the TRIB3 role in specific phenotypes of tumor epithelial cells, the latter of which was exacted from scRNA datasets and further visualized after dimensionality reduction. Notably, the TRIB3^high^ subset was presented in scattered tumor epithelial cells (**Figure 6D**) and shared a much higher resemblance to PT (*R* = 0.864) (**Figure 6E**). In addition, the pseudotime analysis indicated that such a cell subset could be a primitive cancer stem cell (**Figure 6F**) as evidenced by the relatively high expression of the cancer stem cell biomarker CD44 (**Supplementary Figure S7**) (26). The cell–cell communication analysis suggests that TRIB3^high^ tumor cells interact more extensively and strongly than other cell types, particularly for interactions with Mac and T cells (**Figure 6G**, **Supplementary Figure S8**). At the same time, the high expression of glyceraldehyde-3-phosphate dehydrogenase (GAPDH) may predict a strong exosome assembly and aggregation capacity for this cell type (**Supplementary Figure S7**) (27). The results of the cell–cell interaction network analysis disclosed the higher interactions of TRIB3^high^ tumor epithelial cells with other cell types in certain ligand–receptor pairs, spanning CD70-CD27, CLEC2B-KLRB1, CD99-CD99, COL6A2-CD44, COL6A2-SDC4, PGD-VEGFR1, and PROS1-AXL, suggesting that the TRIB3^high^ subset showed stronger local interactions with other major cell types (**Supplementary Figure S9**), which could predispose to an increased ability of induction and reprogramming of extrinsic phenotypic features, thereby reshaping the overall TME. TRIB3^high^ tumor epithelial cells were mainly enriched in apoptosis, ferroptosis, ribosome, and lysosome signaling pathways compared with other cell clusters (**Figures 7A, B**). Lastly, the spatial transcriptomic analysis confirmed that the TRIB3^high^ subset is highly enriched in tumor tissues (**Figure 7C**).

## Discussion

PCDs are fundamental and intricate biological processes in various physiological and pathological events. Evidence persuasively denotes that PCDs are critical regulators in cancer development and progression (28, 29), and key factors in various PCDs have been progressively appreciated, thus applying them in the identification of tumor diagnosis and treatment (30–33). Many association studies between KIRC and PCD have emerged in recent years. However, the different types of PCDs are compartmentalized studies, and there is a dearth of pertinent, comprehensive investigations, particularly in KIRC. In this study, we discovered significant alterations in the CDRG expression levels in KIRC tissues. Such changes are not merely present in the tumor epithelial cell emphasized by traditional studies; similar shifts were also observed in other structural cells and immune cells in the TME, most of which belong to autophagy, apoptosis, and ferroptosis. The landscape of diagnostic and therapeutic targets for PCD, as indicated by *in vitro* and *in vivo* data, continues to evolve, making this an unquestionably fruitful area of research. Ma et al. substantiated that silibinin could induce apoptosis by inhibiting the mTOR-GLI1-BCL2 pathway, thus markedly suppressing the tumor growth of RCC (34), with an *in vitro* cell line assay indicating that capsaicin pronouncedly inhibited the migration and invasion of RCC by inducing autophagy through the AMPK/mTOR pathway (35). Similarly, Heiker et al. clarified that silencing the enzymes essential for the biosynthesis of glutathione or glutathione peroxidase could initiate ferroptosis, thus selectively compromising the KIRC cells’ viability without any impact on the growth of non-malignant renal epithelial cells (36). The results mentioned above noted that PCDs are highly coordinated and regulate the cells’ survival state through various signaling pathways, suggesting its potential as a therapeutic target for RCC.

Currently, the preoperative diagnosis of KIRC heavily relies on MRI/CT. Despite specific enhancement modes for KIRC, misdiagnosis consistently happens in clinical settings (37), imposing a socio-economic burden on healthcare systems globally. Myriads of studies have identified novel diagnostic biomarkers for kidney cancer, spanning long non-coding RNAs, circulating tumor DNA, and circulating tumor cells; despite this, there is still scope for improvement in specificity as well as sensitivity, and the clinical applicability of such emerging biomarkers remains to be further validated (38–41). It is inspiring that blood platelet and blood-derived exosome-based polygenic models manifested excellent diagnostic efficacy, offering an accessible complement to existing screening modalities (42–44). Exosomes are secreted extracellularly by cytosolic fusion with the plasma membrane, which plays an imperative role in shaping the TME (45). Due to the nature of exosomes in mediating intercellular communication and extensive existence in body fluids (*e*.*g*., blood, saliva, and urine), they become an optimal surrogate in cancer diagnosis and therapeutic predictions, also presenting encouraging results in clinical application (46–48). Wang et al. found that tumor cells can reduce T cell activity by secreting exosomal PD-L1 and that exosome inhibitors and ferroptosis inducers can effectively counteract these characteristics and create tumor-specific immunity (25). Zhang and colleagues elucidated that adenosine activation of AKT and ERK signaling mediated by exosome secreted by mesenchymal stem cells could contribute to the facilitation of cartilage repair, thereby reducing apoptosis and modulating immune responses (49). These findings demonstrate that PCDs and exosomes are inextricably linked, indicating that further exploration of the reciprocal activity of PCDs and exosomes in the TME could be employed as a unique avenue for future research into the KIRC pathogenesis.

Based on the transcriptome profiling of blood-derived exosomes from KIRC patients, combined with transcriptomic information from the TCGA KIRC cohort and scRNA-seq data of KIRC, we further screened out the candidate biomarker genes among CRDGs by their correlation with clinical indicators, thus uncovering 13 essential genes with diagnostic potential for KIRC. Using machine learning and their cross-validation, the construction of diagnostic models with 13 key genes showed high diagnostic efficacy in both blood-derived exosome samples and tissue samples, with AUC of 0.965 for blood-derived exosomes and AUC = 0.914 for tissue. The traceability analysis based on single-cell omics showed that the expression and alterations of key genes presented in multiple cellular identities in TME, especially in structural cells and macrophages. TME is a highly heterogeneous ecosystem constituted by cancer cells, fibroblasts, adipocytes, endothelial cells, mesenchymal stem cells, and extracellular matrix (45, 50). Notably, cancer cells could secrete exosomes to induce the production of cancer-associated fibroblasts and cancer-associated endothelial cells, thereby contributing to the remodeling of TME (51–53). Comparably, stromal cells are competent in tumor progression by stimulating and reprogramming cancer cells through exosomes (54, 55). From a theoretical perspective, our studies could accelerate the understanding of the identification of a cancer biomarker, simultaneously facilitating the biological interpretation of cancer biology in the multi-omic context.

Our study noted that the high TRIB3 expression, in one of the genes in the 13-gene diagnostic model, was closely linked with advanced clinical stage and worse prognosis in KIRC patients, which is consistent with the findings of Hong et al., collectively revealing its essential role in KIRC development and progression (56). Meanwhile, the relatively elevated chromatin accessibility of TRIB3 in tumor epithelial cells was manifested in the scATAC data. At the same time, the stRNA-seq verified that TRIB3 was predominantly expressed in cancer tissues, further justifying its upregulated expression pattern in KIRC. The biological role of TRIB3 is extensive. In addition to being associated with ferroptosis (57), the upregulation of TRIB3 could suppress the process of autophagy (58, 59). Furthermore, TRIB3 is implicated in the carcinogenesis of a variety of cancers, with evidence indicating that it could inhibit the degradation of FOXO1 and enhance SOX2 transcription, thus contributing to the carcinogenesis of breast cancer (60) and induction of immune evasion by inhibiting the STAT1–CXCL10 axis and impeding the CD8+ T cell infiltration in colorectal cancer (61). Intriguingly, its relationship with exosomes has also been investigated, indicating that TRIB3 could mediate the impairment of autophagy and facilitate the secretion of INHBA/Activin A-enriched exosomes of hepatocellular carcinoma, thus resulting in the occurrence of liver fibrosis (59). On this basis, our further analysis of the TRIB3^high^ subset revealed that such cell subtype interacts more extensively and strongly than the other cell types, representing an optimized remodeling of the TME and maintaining tumor progression. Functionally, the TRIB3^high^ tumor epithelial cell was highly enriched in ribosomes and PCD-related pathways, representing its high metabolic demand, while its high expression of CD44 suggests a high degree of stemness. Such discoveries were validated in a study by Hua et al., elucidating that TRIB3 interacts with β-catenin and TCF4 in intestinal cells, thereby increasing the expression of cancer stem cell-related genes (62). Meanwhile, it was shown that a high expression of the GAPDH plays a facilitating role in the assembly and secretion of exosomes by cells (27), which is consistent with the TRIB3^high^ tumor epithelial cells, and this is probably a potential mechanism for the regulation of TME of such subset.

To the best of our knowledge, the present study portrays the first landscape of PCDs in KIRC and further explores the identified biomarkers’ diagnostic role and biological functions. Nevertheless, our study still has some unavoidable shortcomings. First, the diagnostic model needs to be further validated by expanding the validation cohort; second, additional experimental tools are needed further to investigate the physiopathological mechanisms of the relevant molecules; and finally, the therapeutic potential of such biomarkers remains to be further elucidated. In conclusion, the exosome is an essential mechanism to determine cell fate in addition to cell surface ligand–receptor interaction, which could be the game-changer in shaping the TME. In this study, we constructed a diagnostic model based on PCD-related genes. Furthermore, we validated the diagnostic efficacy in multiple KIRC cohorts, subsequently exploring the mechanism through single-cell omics, thus providing a novel perspective for the early diagnosis of KIRC and facilitating the understanding of the mechanisms of KIRC.

## Data availability statement

The datasets presented in this study can be found in online repositories. The names of the repository/repositories and accession number(s) can be found within the article/**Supplementary Material**.

## Ethics statement

Ethical review and approval was not required for the study on human participants in accordance with the local legislation and institutional requirements. Written informed consent for participation was not required for this study in accordance with the national legislation and the institutional requirements.

## Author contributions

CW and SC designed this work. CW, YH, and JZ integrated and analyzed the data and wrote this manuscript. CW, XW, and SC edited and revised the manuscript. All authors contributed to the article and approved the submitted version.

## Supplementary material

Supplementary Figure 1 Quality control (QC) for scRNA-seq data. **(A)** Violin plot showing the number of genes, unique molecular identifiers (UMIs), and the percentage of mitochondrial genes of each sample before QC. **(B)** Violin plot showing the number of genes, UMIs, and the percentage of mitochondrial genes of each sample after QC.

Supplementary Figure 2 Single-cell RNA sequencing (scRNA-seq) profiling of kidney renal clear cell carcinoma (KIRC). **(A)** Uniform manifold approximation and projection (UMAP) plot presenting the cell clusters of scRNA-seq. **(B)** UMAP plots showing the data sources of scRNA-seq. **(C)** UMAP plots presenting the 17 cell clusters of scRNA-seq.

Supplementary Figure 3 Kyoto Encyclopedia of Genes and Genomes (KEGG) pathway enrichment analysis of differentially expressed genes (DEGs) between different datasets. **(A)** KEGG pathway enrichment for upregulated DEGs. **(B)** KEGG pathway enrichment for downregulated DEGs. The visualized results were the top 30 intersectional enriched terms of the pathways between the datasets. The red bubble represents the enrichment terms of upregulated DEGs (left), and the blue bubble represents the enrichment terms of downregulated DEGs (right). Shades of color in the bubble indicate negative log10(*P*-value), and the bubble sizes indicate the number of genes enriched in the pathway.

Supplementary Figure 4 Screen for the candidate biomarker genes. **(A)** Bubble plots showing the 33 candidate biomarker genes that were downregulated in the exoRBase kidney renal clear cell carcinoma (KIRC) cohort and their expression pattern in other datasets. Red circles represent positive logFC values or upregulated DEGs in the corresponding datasets, while blue circles represent positive logFC values or downregulated DEGs in the corresponding datasets. The bubble size indicates negative log10(*P*-value). **(B)** The survival analysis results indicated that 21 candidate biomarker genes were significantly associated with the overall survival of KIRC cases based on the GEPIA database.

Supplementary Figure 5 Correlation analysis of the clinical stage of kidney renal clear cell carcinoma patients for candidate biomarker genes. The patients were grouped according to stages I–IV. The differences in the expression of candidate biomarker genes were compared between groups, and *P <*0.05 genes were retained.

Supplementary Figure 6 A total of 13 genes in 10 main cell clusters. Violin plot showing the differential analysis of key genes in various cell clusters.

Supplementary Figure 7 Violin plot showing the differential analysis of CD44 and GAPDH in tumor cells *versus* TRIB3^high^ tumor epithelial cells.

Supplementary Figure 8 Analysis of cell–cell signal interaction pathway networks for cell clusters. Circos plot showing putative ligand–receptor interactions between each cell cluster, with the weight of interactions indicated by the thickness of the connecting lines.

Supplementary Figure 9 Bubble plot of tumor cell ligand–receptor interactions in the tumor microenvironment. Summary of selected ligand–receptor interactions between different cell clusters between TRIB3^high^ tumor epithelial cells and other cell types. The *P*-values are indicated by the size of each circle. In contrast, the color gradient indicates the level of interaction.
