# Supplementary figures and images for: Dissecting order amidst chaos of programmed cell deaths: construction of a diagnostic model for KIRC using transcriptomic information in blood-derived exosomes and single-cell multi-omics data in tumor microenvironment
Source: Front Immunol. 2023 Apr 19;14:1130513. doi: 10.3389/fimmu.2023.1130513 (PMC10154557; doi:10.3389/fimmu.2023.1130513)

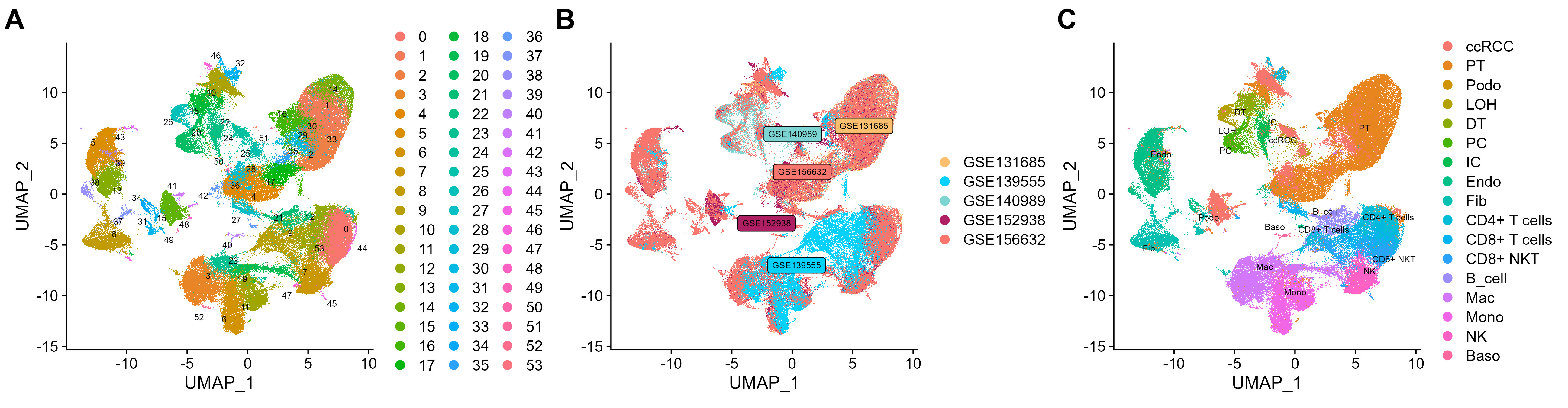

Supplement: Supplementary Figure 2 — Single-cell RNA sequencing (scRNA-seq) profiling of kidney renal clear cell carcinoma (KIRC). (A) Uniform manifold approximation and projection (UMAP) plot presenting the cell clusters of scRNA-seq. (B) UMAP plots showing the data sources of scRNA-seq. (C) UMAP plots presenting the 17 cell clusters of scRNA-seq. [file Image_2.tif]

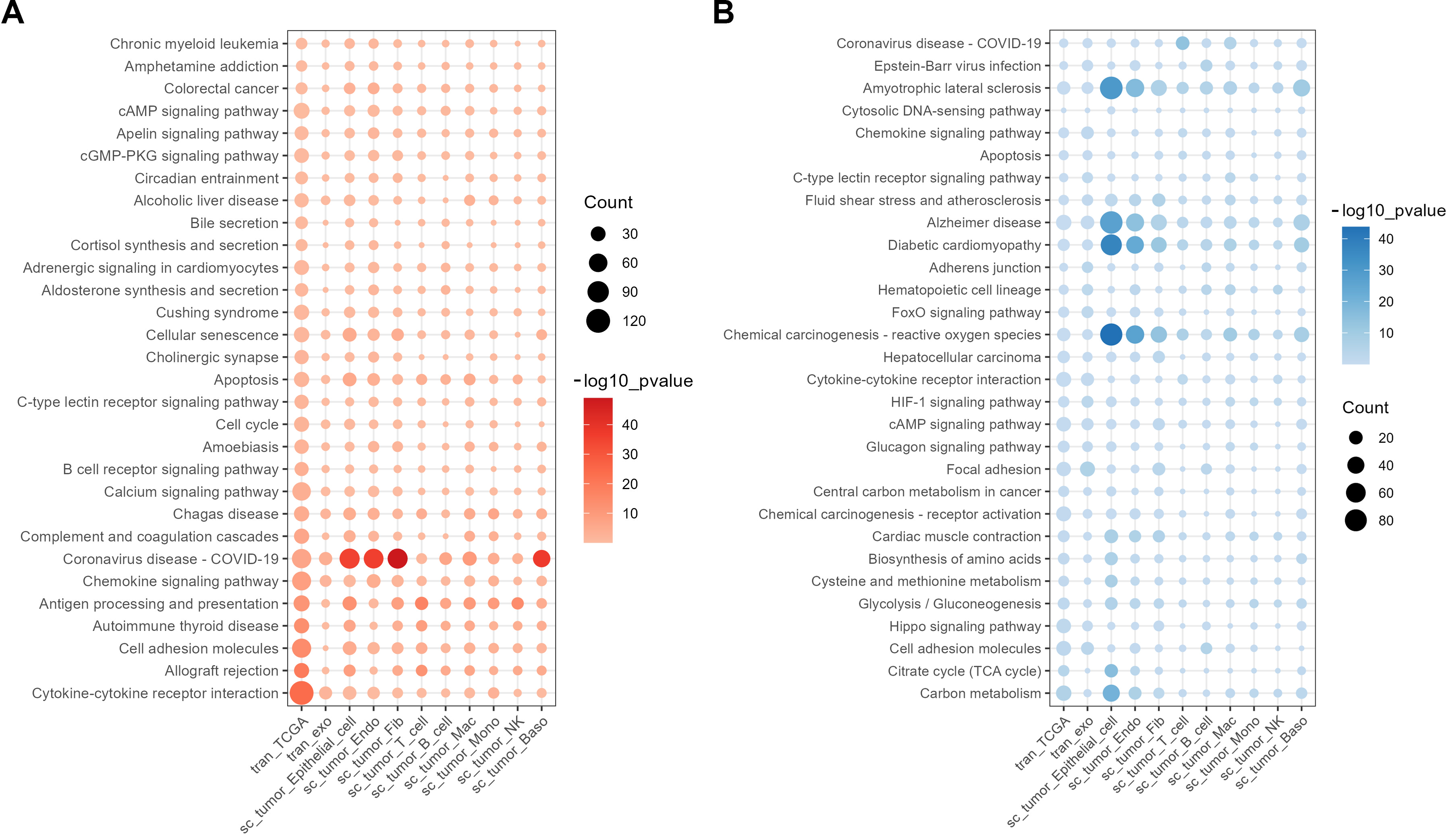

Supplement: Supplementary Figure 3 — Kyoto Encyclopedia of Genes and Genomes (KEGG) pathway enrichment analysis of differentially expressed genes (DEGs) between different datasets. (A) KEGG pathway enrichment for upregulated DEGs. (B) KEGG pathway enrichment for downregulated DEGs. The visualized results were the top 30 intersectional enriched terms of the pathways between the datasets. The red bubble represents the enrichment terms of upregulated DEGs (left), and the blue bubble represents the enrichment terms of downregulated DEGs (right). Shades of color in the bubble indicate negative log10(P-value), and the bubble sizes indicate the number of genes enriched in the pathway. [file Image_3.tif]

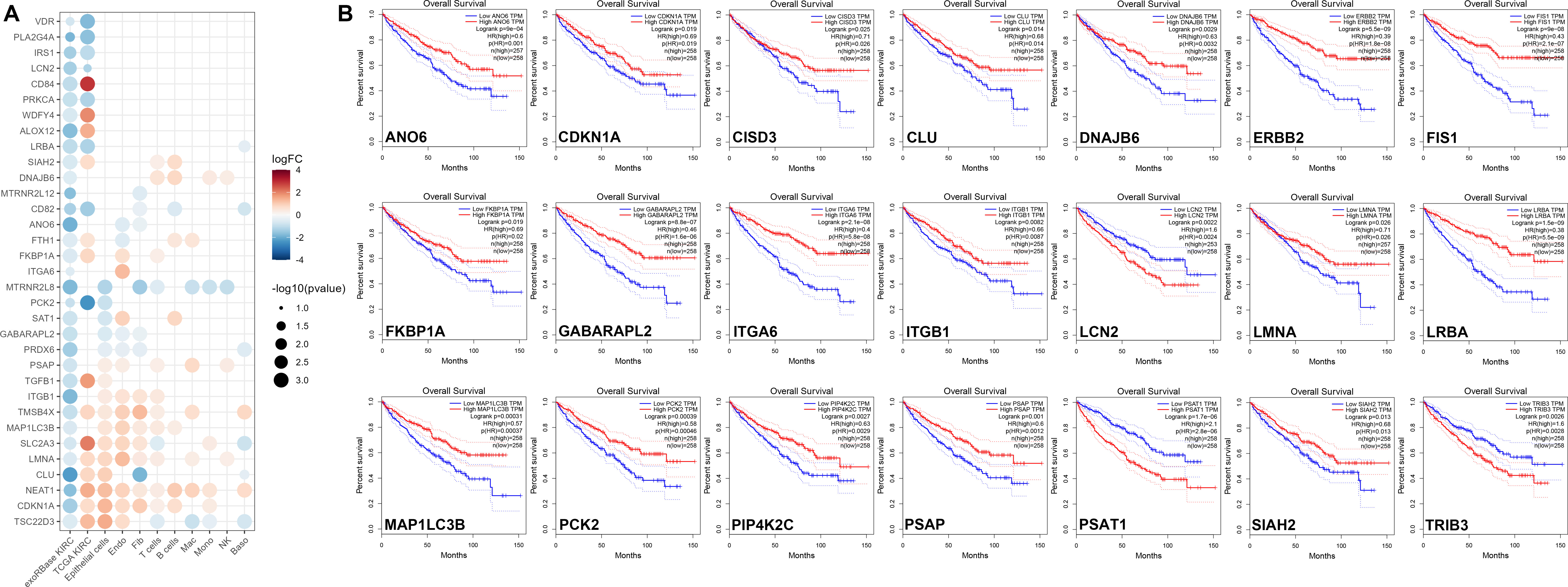

Supplement: Supplementary Figure 4 — Screen for the candidate biomarker genes. (A) Bubble plots showing the 33 candidate biomarker genes that were downregulated in the exoRBase kidney renal clear cell carcinoma (KIRC) cohort and their expression pattern in other datasets. Red circles represent positive logFC values or upregulated DEGs in the corresponding datasets, while blue circles represent positive logFC values or downregulated DEGs in the corresponding datasets. The bubble size indicates negative log10(P-value). (B) The survival analysis results indicated that 21 candidate biomarker genes were significantly associated with the overall survival of KIRC cases based on the GEPIA database. [file Image_4.tif]

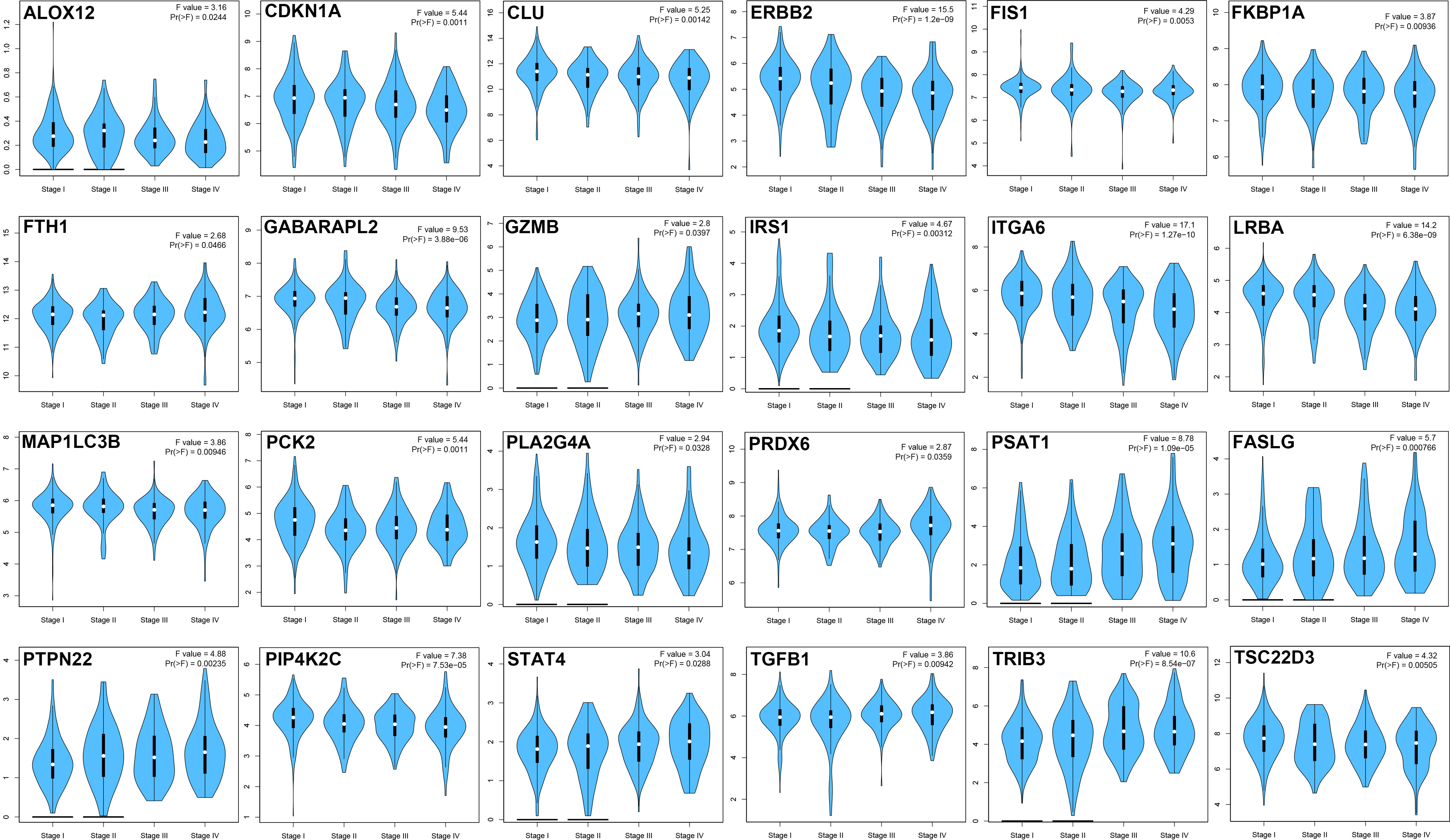

Supplement: Supplementary Figure 5 — Correlation analysis of the clinical stage of kidney renal clear cell carcinoma patients for candidate biomarker genes. The patients were grouped according to stages I–IV. The differences in the expression of candidate biomarker genes were compared between groups, and P <0.05 genes were retained. [file Image_5.tif]

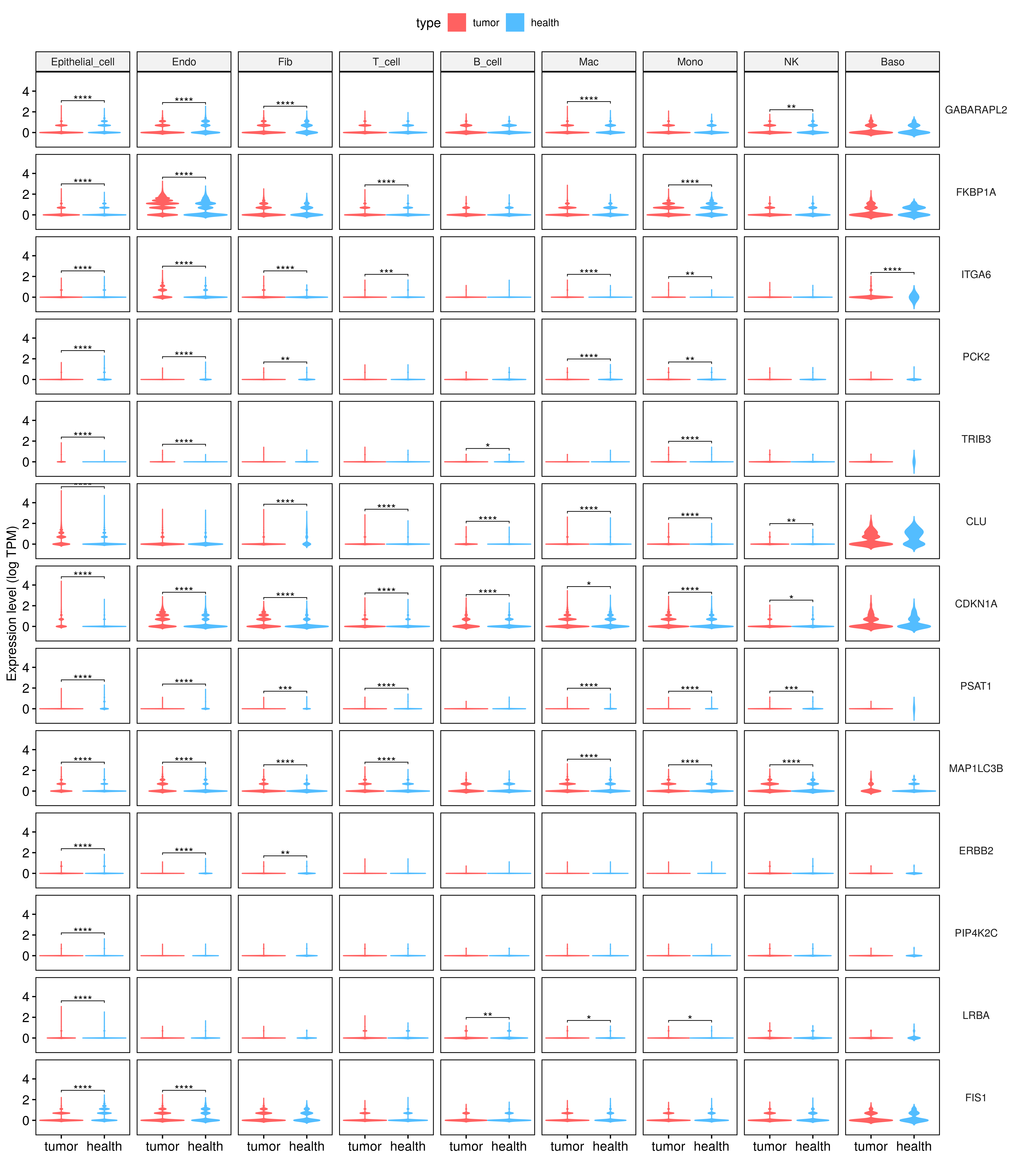

Supplement: Supplementary Figure 6 — A total of 13 genes in 10 main cell clusters. Violin plot showing the differential analysis of key genes in various cell clusters. [file Image_6.tif]

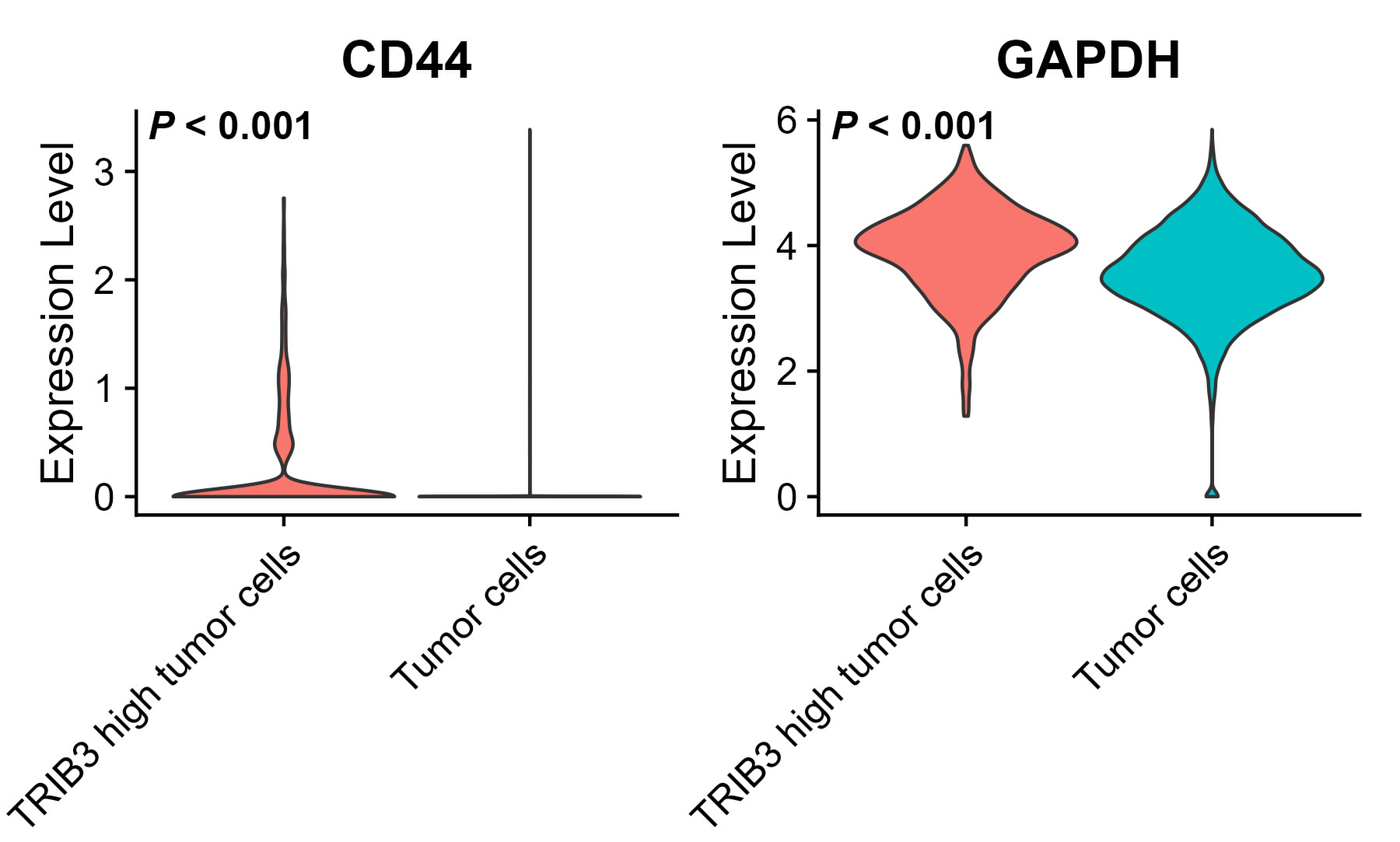

Supplement: Supplementary Figure 7 — Violin plot showing the differential analysis of CD44 and GAPDH in tumor cells versus TRIB3high tumor epithelial cells. [file Image_7.tif]

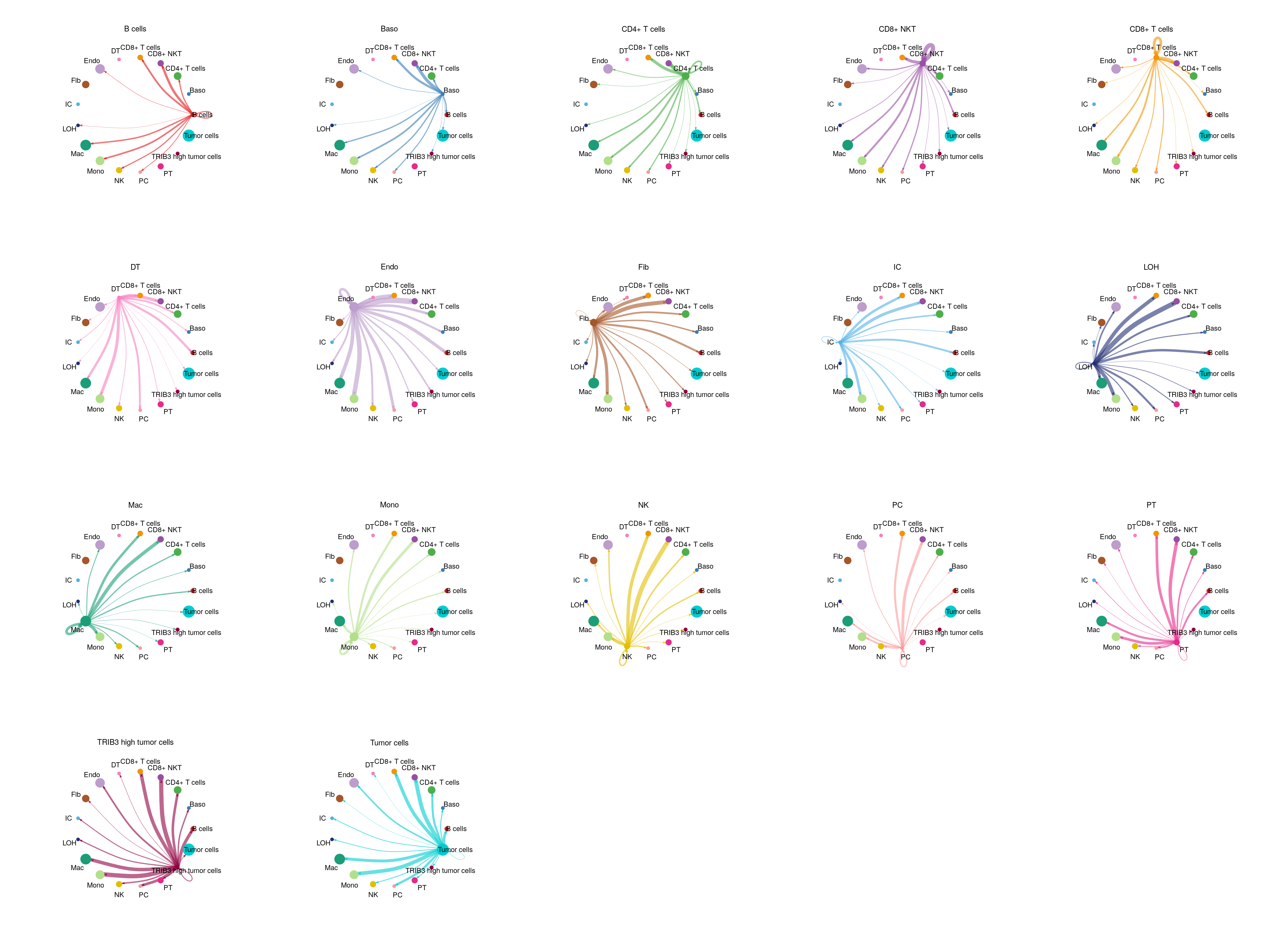

Supplement: Supplementary Figure 8 — Analysis of cell–cell signal interaction pathway networks for cell clusters. Circos plot showing putative ligand–receptor interactions between each cell cluster, with the weight of interactions indicated by the thickness of the connecting lines. [file Image_8.tif]

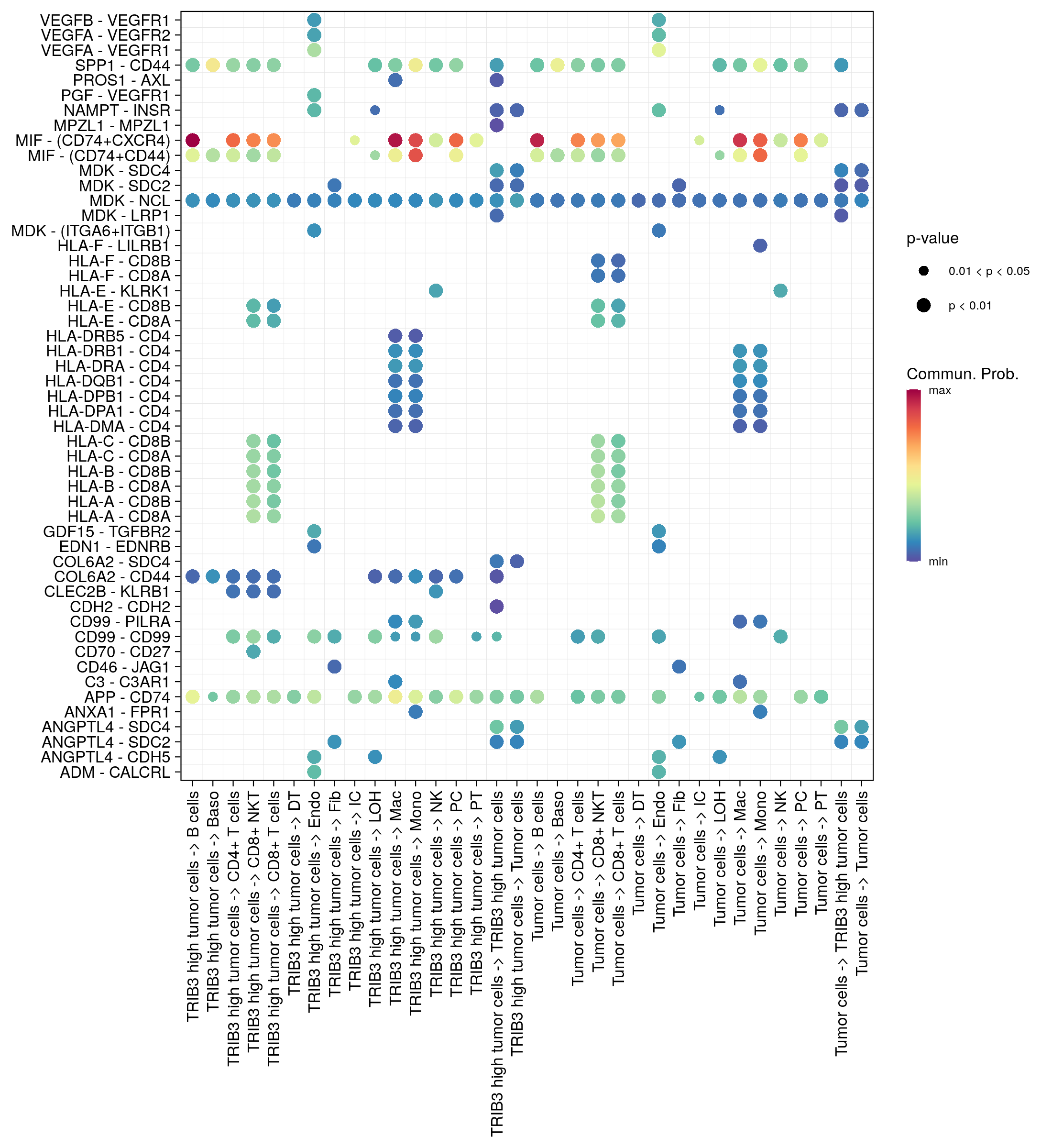

Supplement: Supplementary Figure 9 — Bubble plot of tumor cell ligand–receptor interactions in the tumor microenvironment. Summary of selected ligand–receptor interactions between different cell clusters between TRIB3high tumor epithelial cells and other cell types. The P-values are indicated by the size of each circle. In contrast, the color gradient indicates the level of interaction. [file Image_9.tif]
